# Influence of GaAsBi Matrix on Optical and Structural Properties of InAs Quantum Dots

**DOI:** 10.1186/s11671-016-1470-1

**Published:** 2016-06-02

**Authors:** Peng Wang, Wenwu Pan, Xiaoyan Wu, Juanjuan Liu, Chunfang Cao, Shumin Wang, Qian Gong

**Affiliations:** State Key Laboratory of Functional Materials for Informatics, Shanghai Institute of Microsystem and Information Technology, Chinese Academy of Sciences, Shanghai, 200050 China; School of Physics, University of Chinese Academy of Sciences, 19 Yuquan Road, Beijing, 100049 China; Department of Microtechnology and Nanoscience, Chalmers University of Technology, 41296 Gothenburg, Sweden

**Keywords:** InAs, Quantum dot, GaAsBi, MBE, Thermal stability

## Abstract

InAs/GaAsBi dot-in-well structures were fabricated using gas-source molecular beam epitaxy and investigated for its optical and structural properties. GaAsBi-strained buffer layer and strain reduction layer are both effective to extend the photoluminescence (PL) emission wavelength of InAs quantum dot (QD). In addition, a remarkable PL intensity enhancement is also obtained compared with low-temperature-grown GaAs-capped InAs QD sample. The GaAsBi matrix also preserves the shape of InAs QDs and leads to increase the activation energy for nonradiative recombination process at low temperature. Lower density and larger size of InAs QDs are obtained on the GaAsBi surface compared with the QDs grown on GaAs surface.

## Background

Semiconductor quantum dots (QDs) are promising candidates for a variety of optoelectronic applications, including lasers, photovoltaic devices, and photon detectors [1]. In particular, self-assembled InAs/GaAs QDs laser has been studied for decades as its great potential for optical fiber communication at 1.31 μm [2]. Room temperature (RT) continuous-wave (CW)-operated InAs QD laser with emission wavelength 1.31 μm have been fabricated on silicon (Si) substrate by using germanium (Ge; only 0.08 % mismatched with GaAs) as an intermediate layer, which is very significant for the realization of Si-based photoelectric integration [3–5]. The active region is based on the so called dot-in-well (DWELL) structure where the dots are embedded in a matrix with the form of a quantum well (QW). Optical and structural properties of the InAs QDs strongly depend on the embedded matrix which is splitted into two parts by the dot layer, i.e., the strain reduction layer (SRL) on top and the strained buffer layer (SBL) at the bottom of the dot layer. InGaAs matrix has been most commonly utilized in the DWELL structure for the fabrication of InAs QD lasers [6]. As many previous studies revealed, the InGaAs SRL reduces the inner compressive strain of InAs QD, leading to a large red-shift of PL emission, while the InGaAs SBL increases the InAs QD density, leading to a remarkable emission intensity enhancement [7, 8]. InAlAs cap layer has also been utilized as the SRL, which are effective to prevent the segregation of In atoms and increase the energy difference between the ground and excited level of electrons in InAs QD [9, 10]. In addition, Liu et al. had investigated the influence of GaAsSb cap layer on the optical and structural properties of the InAs QD, and found that dot decomposition during capping was suppressed and type-II band structure was formed, indicated by a significant blue-shift of the PL emission [11, 12]. InAs QD capped with quaternary compound materials has also been studied. Mamutin et al. had extended the emission wavelength of InAs QD above 1.7 μm by using InGaAsN matrix [13], and Keizer et al. had investigated the morphology and PL characteristics of InAs QD after capped with a GaAsNSb thin layer [14]. Therefore, the properties of InAs QD embedded in a novel matrix should always be worth to study. On the other hand, dilute bismide GaAsBi has attracted great attentions recently, due to its promising properties such as large band-gap bowing effect, band-gap temperature insensitivity, and large spin-orbit splitting [15]. Furthermore, GaAsBi has lattice constant slightly larger than GaAs, and, is naturally expected as a very good candidate for the SRL and SBL layer of the InAs QDs. The low-temperature growth nature of GaAsBi might also prevent the intermixing of In/Ga. However, there are no studies published on the growth of InAs QD in the GaAsBi matrix so far.

In this work, we have grown InAs/GaAsBi DWELL samples on semi-insulating (SI) GaAs (100) substrate by gas-source molecular beam epitaxy (GS-MBE). The influence of GaAsBi matrix on the optical and structural properties of InAs QDs has been studied. Along with a remarkable extending effect for the PL emission wavelength, the GaAsBi matrix also improves the thermal property and size uniformity of InAs QDs, which make GaAsBi a proper low-temperature-grown embedding matrix for QD in the DWELL structure. In addition, the GaAsBi cap layer plays an important role in preserving the QD shape and suppressing the In/Ga intermixing.

## Methods

InAs/GaAsBi DWELL samples were grown on SI GaAs (100) substrate by VG V90 GS-MBE. The entire grown structure was started with a 100-nm GaAs buffer layer grown at 580 °C after desorption of the native oxide layer. Then the substrate temperature (*T*_s_) was lowered for the growth of DWELL structure, following which, the *T*_s_ was raised back to 580 °C for the deposition of 100 nm GaAs cladding layer. For morphology analysis, InAs QD deposited on the designed SBL was repeated on the surface. The growth temperature for the InAs QD layer and InGaAs SBL were 500 °C. The GaAsBi matrix was grown at 400 °C. Five different DWELL structures were specially designed as shown in Table 1. For comparison, three different types of under layer were chosen, i.e., GaAs, In_0.1_Ga_0.9_As, and GaAs_0.97_Bi_0.03_, respectively. InAs dot layer with nominal 2.2 monolayers (MLs) was grown on the under layer and then capped by the capping layer (GaAs_0.97_Bi_0.03_ or GaAs). The deposition rate of InAs QDs layer is 0.1 ML/s for all samples. The growth temperatures and embedding matrix structures are listed in Table 1.

**Table 1:** InAs DWELL sample list with different matrix designs

Samples	Under layer	InAs QDs	Capping layer
(a)	2 nm GaAs_0.97_Bi_0.03_ SBL(400 °C)	2.2 MLs	6 nm GaAs_0.97_Bi_0.03_ SRL (400 °C)
(b)	GaAs(500 °C)	2.2 MLs	6 nm GaAs_0.97_Bi_0.03_ SRL (400 °C)
(c)	GaAs(500 °C	2.2 MLs	GaAs (400 °C)
(d)	GaAs(500 °C	2.2 MLs	GaAs (500 °C)
(e)	2 nm In_0.1_Ga_0.9_As SBL(500 °C)	2.2 MLs	6 nm GaAs_0.97_Bi_0.03_ SRL (400 °C)

Photoluminescence (PL) spectra were measured with a Nicolet Magna 860 Fourier transform infrared spectrometer (FTIR). A liquid-nitrogen cooled InSb detector and a CaF_2_ beam splitter were equipped. Samples were excited by a diode-pumped solid state (DPSS) laser (*λ* = 532 nm), and the double modulation mode was used to eliminate the mid-infrared background radiation over 2 μm. Temperature-dependent PL measurements were carried out by mounting samples into a continuous-flow helium cryostat, and a Lake Shore 330 temperature controller was used to adjust the temperature form 8 to 250 K. The laser spot area is 4.5 × 10^−2^ cm^2^. The laser power used in RT and temperature-dependent PL measurements are 9.4 and 542.3 mW, respectively. The InAs QD morphology was measured by a Bruker Icon atom force microscope (AFM) in the tapping mode. The scanned area was 2 × 2 μm^2^ square. Flooding analysis was carried out to record the size distribution of the surface InAs QDs. Energy dispersive X-ray (EDX) mapping measurement of surface and embedded InAs QDs were also carried out for direct investigation of InAs QD capped by GaAsBi SRL.

## Results and Discussions

**Fig. 1:**
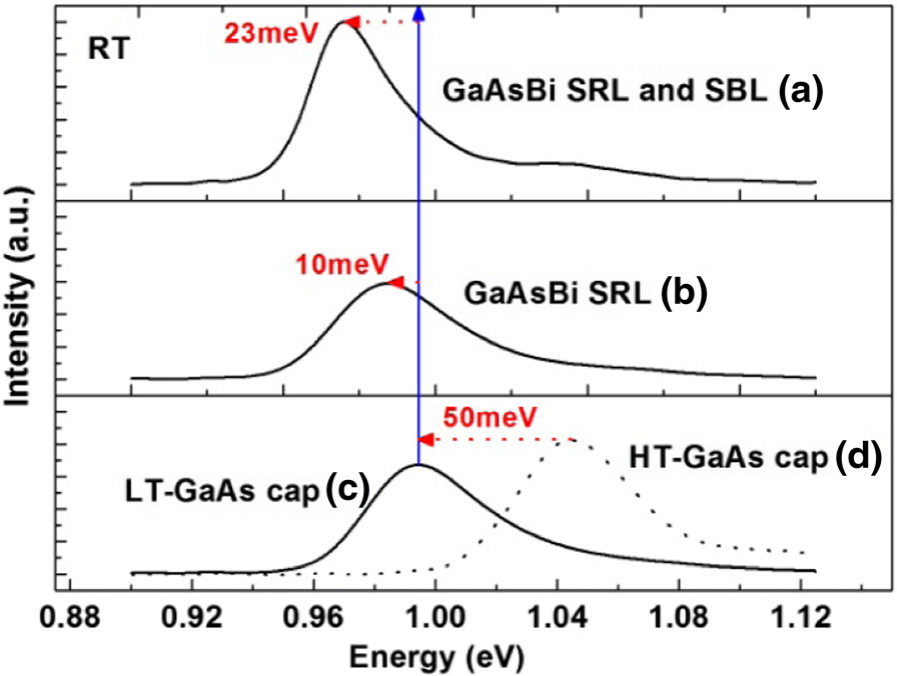
Room-temperature PL spectra embedded in different matrix

Firstly, we investigated the influence of GaAsBi SBL and SRL on the PL emission wavelength and the PL intensity of InAs QDs. Two reference samples were grown with the InAs QDs capped by GaAs, at low-growth temperature (LT; c) and high growth temperature (HT; d), respectively. RT PL spectra of the DWELL structures were shown in Fig. 1. Strong emissions at near-infrared band were observed for all samples. Ground state (GS)-related emissions are verified to dominate all the spectra. Compared to sample (d), a red-shift of 50 meV and a slight intensity decrease are observed in the PL spectrum of sample (c) where the InAs QDs is capped with LT grown GaAs cap. Previous study has reported that InAs QD preserved larger size when capped at LT because of the suppressed dot decomposition [16], which is also confirmed by the EDX mapping measurement latter in this paper. This might be the reason for the 50-meV red-shift mentioned above. If the LT-grown GaAs in sample (c) is replaced by a GaAsBi SRL, a further red-shift of 10 meV is observed, as shown by the PL spectrum of sample (b) in Fig. 1. Furthermore, when a GaAsBi SBL is grown as in sample (a), an additional red-shift of 13 meV is obtained. Therefore, both the GaAsBi SRL and SBL make contribution to extend the emission wavelength of the InAs QD. It is worth noting that the emission intensity of sample (a) increased by 1.5 times than that of sample (c), which is a very desirable optical property. Moreover, the PL linewidths at 8 K are 35.33, 55.84, and 50.49 meV for sample (a), sample (c), and sample (d), indicating that the GaAsBi SBL and SRL might improve the uniformity of the InAs QDs.

**Fig. 2:**
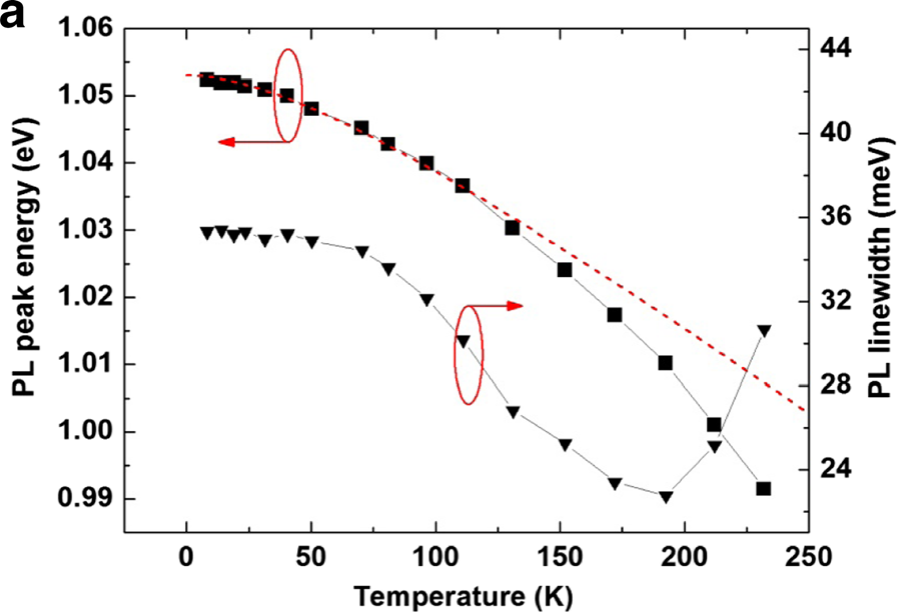
PL peak energy and linewidth of sample (**a**) as a function of temperature

For further investigation of the optical properties of InAs/GaAsBi DWELL structure, we have carried out the temperature-dependent PL measurement. The results are shown in Fig. 2. The peak energy decreasing with temperature exactly follows the InAs band gap variation (red dashed curve) in the temperature range from 0 up to about 110 K. When the temperature is raised further, the PL peak energy of QD decreases faster than that predicted by the empirical Varshni law. This is due to the thermally activated redistribution of carriers between the QDs. Gradually enhanced occupation of carrier in the large dots, which has lower ground state energy, leads to faster emission energy decreasing. The temperature dependence of PL linewidth shown in Fig. 2 (inverted triangle symbols) can be explained by the same reason. For temperature lower than 50 K, the linewidth is almost constant at about 35 meV. Then, the PL linewidth decreases in the temperature range from 50 to 190 K, followed by a remarkable increase when the temperatures are above 190 K. In the low temperature range, the carriers have no enough kinetic energy to transfer between dots, leading to a non-Fermi distribution. All the QDs captured carriers might contribute to the PL signal. The deviation of QD size is reflected by the large PL linewidth. When temperature increases, thermally activated carriers start to escape from the small dots which have small carrier confinement, resulting in a net transfer of carriers from small QDs to large ones. The PL linewidth, thus, decreases. When the temperatures are above 190 K, the carriers captured in all the QDs (small and large) might gradually escape from the QD confinement due to the thermal activation process, resulting in carrier redistribution in all the QDs and the PL linewidth increases again. This behavior is similar to the results previously reported in the InAs/GaAs DWELL structure [17].

**Fig. 3:**
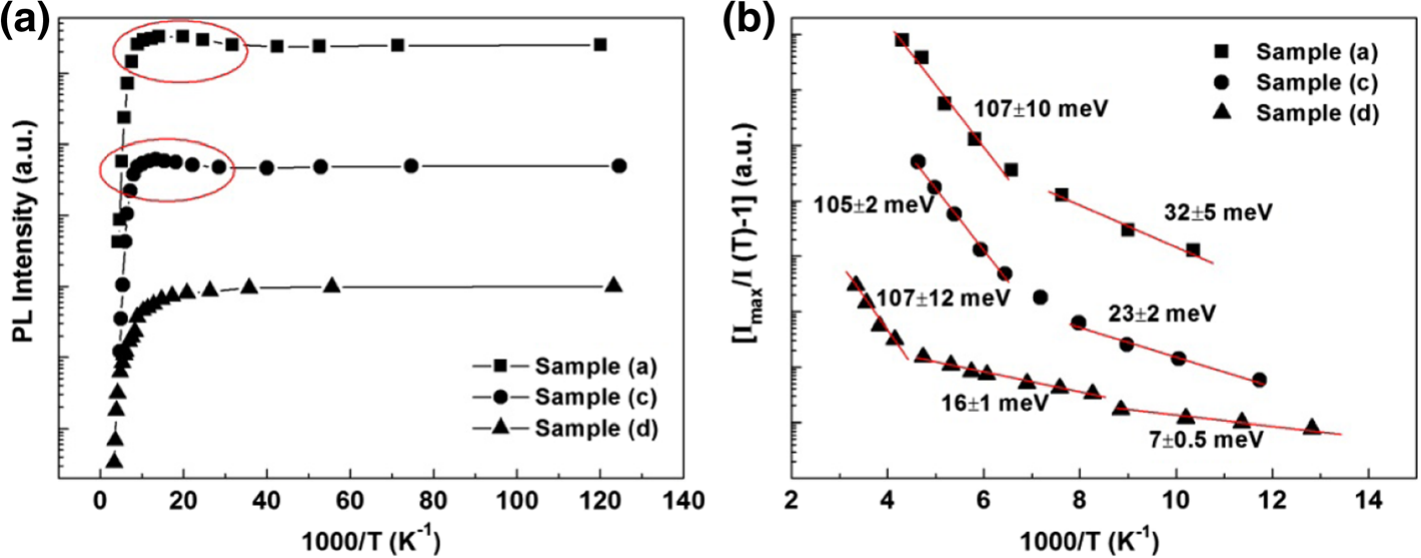
**a** PL intensity thermal quenching behavior and **b** activation energy fitting in the Arrhenius plot

The integrated PL intensity of sample (a) is shown as a function of temperature in Fig. 3a, where the results of sample (c) and sample (d) are also presented for comparison. Two regions are revealed for all the three samples: below 70 K, the integrated PL intensity almost remain unchanged, and at the higher temperature, they decrease dramatically with the increasing temperature. This quenching of PL is usually explained by thermal escape of the carriers from QDs to the surrounding matrix. It is worth noting that a significant PL intensity increasing is observed for both sample (a) and sample (c) in the region marked by the red circles. Similar variation behavior is also reported by Popescu et al. [18] and Mu et al. [19]. A potential barrier might exist at the interface between InAs QDs and its surrounding materials which prevents the carriers to be trapped by dots at low temperatures. Increasing the temperature provides thermal energy for the carriers to overcome the potential barrier and makes the carriers trapped more efficiently by the QD, leading to enhancement of the PL. The intensity increasing regions are 26–61 K and 26 75 K for sample (a) and sample (c), respectively. Since the GaAsBi matrix is grown at low temperature, the In/Ga intermix are reduced during the capping process of InAs QDs and a relatively abrupt interface is formed which leads to the potential barrier for the carriers trapping.

In order to study the thermal quenching process of InAs QDs, the integrated PL intensity is plotted versus temperature in the Arrhenius plot shown in Fig. 3b. Equation (1) has revealed the temperature dependence of the integrated PL intensity *I*(*T*), *k* is the Boltzmann’s constant and *E*_*i*_ represents the activation energy of the corresponding thermally activated nonradiative recombination process (NRP). For sample (d), three NRPs are deduced with activation energy *E*_1_ = 7 ± 0.5 meV, *E*_2_ = 16 ± 1.0 meV, and *E*_3_ = 107 ± 12 meV. The lower activation energy *E*_1_ and *E*_2_ is related to the carrier loss to the nonradiative recombination centers such as defect states or due to the QD size inhomogeneity [20]. However, just two NRPs are extracted for sample (a) and sample (c), and the dividing temperature point are both around 140 K. For the NRP above 140 K, the activation energies are 107 ± 10 and 105 ± 2 meV, which are equal to *E*_3_ of sample (d) within the error range. This activation energy around 107 meV should be the energy required for the thermal escape of carrier from the InAs QDs. In terms of the NRP below 140 K, the GaAsBi SBL has raised the activation energy from 23 ± 2 meV to 32 ± 5 meV.

1 $$ I(T) \sim \frac{1}{1+{\mathrm{A}}_1\cdot \exp \left(-\frac{E_1}{k\mathrm{T}}\right)+{\mathrm{A}}_2\cdot \exp \left(-\frac{E_2}{k\mathrm{T}}\right) + \cdot \cdot \cdot } $$

**Fig. 4:**
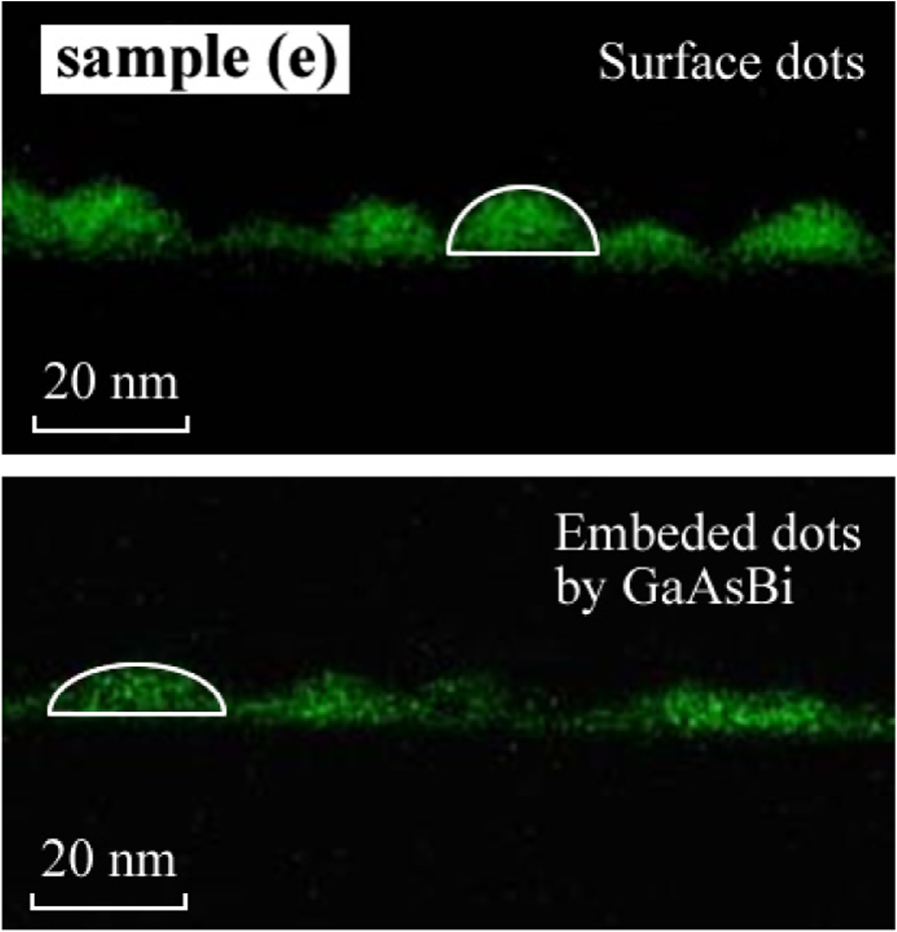
Cross-section EDX mapping of surface and embedded InAs QDs of sample (e)

In order to make a direct investigation to the influence of GaAsBi capping layer on the geometrical shape of InAs QDs, we carried out the EDX mapping measurement of surface and embedded InAs QDs, as shown in Fig. 4. It is found that InAs QDs grown on the surface have a shape of spherical cap, which is preserved when the dot is capped with the GaAsBi capping layer. Our former studies [21] have revealed that the InAs QDs are flattened when the dot is capped by HT-grown GaAs capping layer because of In/Ga intermixing. With the GaAsBi capping layer, the height of InAs QDs decrease about only 23 % which is much lesser than the case of QDs capped with HT-grown GaAs (40 %). Therefore, it is obvious that the LT growth process of GaAsBi SRL has prevented the In/Ga intermixing and left a relative abrupt interface between InAs QD and surrounding matrix, which is consistent with the explanation for the abnormal PL intensity development in Fig. 3a.

**Fig. 5:**
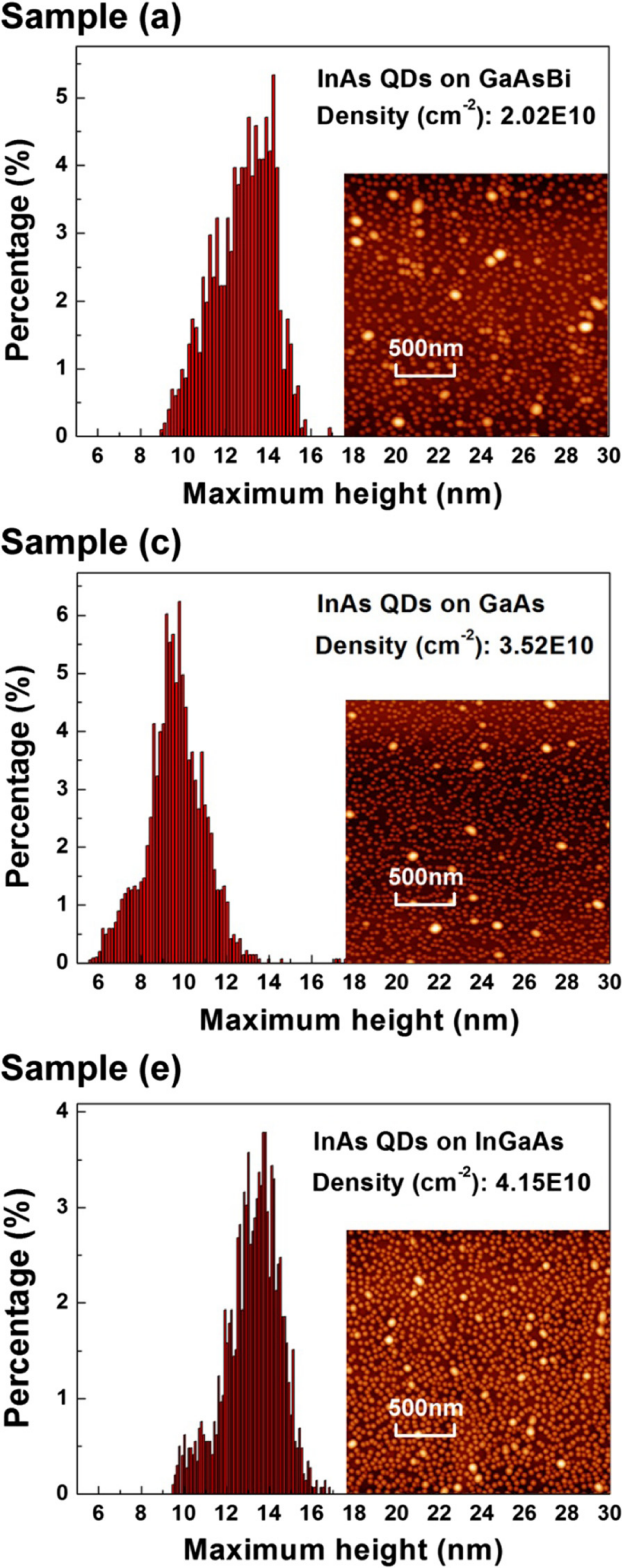
AFM images of InAs QDs assembled on different surfaces and their height distribution

The morphology of InAs QDs grown on different surface is shown by the AFM images in Fig. 5, and the histograms of the height distribution are also plotted. The dot density of QDs grown on GaAsBi has decreased dramatically to 2.02 × 10^10^ cm^−2^, while the dot density is 3.52 × 10^10^ cm^−2^ for QDs grown on GaAs. Both of the decrease in QD density and the increase in QD height reveals a significant enhancement of the In surface diffusion during the growth on the GaAsBi surface. This behavior agrees well with the previous report by Fan et al., which verified the Bi atom as the surfactant during the InAs QD growth [22]. When the InAs QDs are grown on the InGaAs surface, both of the dot density and height increases.

## Conclusions

The optical and structural properties of InAs/GaAsBi DEWLL structure have been investigated. It is found that both the GaAsBi SRL and SBL contribute in extending the PL emission wavelength of InAs QDs. The GaAsBi matrix also helps to decrease the PL linewidth and increase the activation energy of NRP at low temperatures. Lower density and larger size of InAs QDs are obtained on the GaAsBi surface compared with the QDs grown on GaAs surface. The In/Ga intermixing is greatly suppressed when the InAs QDs is capped by GaAsBi. This study has demonstrated that GaAsBi is a good low-temperature-grown embedding matrix for InAs QD in the DWELL structure.

## Abbreviation

AFM: atom force microscope
CW: continuous wave
DWELL: dot-in-well
EDX: energy dispersive X-ray
GS: ground state
GS-MBE: gas-source molecular beam epitaxy
ML: monolayer
NRP: nonradiative recombination process
PL: photoluminescence
QD: quantum dot
QW: quantum well
RT: room temperature
SBL: strained buffer layer
SI: semi-insulation
SRL: strain reduction layer
